# Standardised Suffixes in the Nomenclature of the Higher Taxa of Prokaryotes an Aid to Data Mining, Database Administration and Automatic Assignment of Names to Taxonomic Ranks

**DOI:** 10.1007/s00284-020-01890-y

**Published:** 2020-01-31

**Authors:** B. J. Tindall

**Affiliations:** Leibniz-Institut DSMZ-Deutsche Sammlung Von Mikroorganismen Und Zellkulturen GmbH, Inhoffenstraße 7B, 38124 Braunschweig, Germany

## Abstract

The formation and use of the scientific names of prokaryotes is governed by the International Code of Nomenclature of Prokaryotes. Originally deriving from the 1935 revision of the International Code of Botanical Nomenclature, it retains the treatment of scientific names as Latin words. Above the rank of genus the rank is generally denoted by a single, standardised suffix. This has great advantage in text mining and database infrastructure where the identification of the standardised suffix can automatically be linked to the rank at which the scientific name is being used. The only exception at present are names at the rank of class where, although a standardised suffix has been proposed (-*ia*) it does not allow one to unambiguously identify the rank of the scientific name, since it is also a suffix used at the rank of genus. In addition, due to the fact that the suffix at the rank of class was not regulated in earlier versions of the International Code of Nomenclature of Bacteria, there are names that do not follow the standardised suffix. Uniformity would be an advantage. The problem and a proposed solution are discussed.

## Introduction

The International Code of Nomenclature of Prokaryotes [1] regulates the way the scientific names of prokaryotes are formed and used. Initially covered by the International Code of Botanical Nomenclature, a separate Code of Nomenclature for bacteria (prokaryotes) was formulated by making comparisons with the Entomological Code, the International Rules of Zoological Nomenclature and the International Rules of Botanical Nomenclature [2], with the text from the 1935 revision of the International Rules of Botanical Nomenclature (the Cambridge Code) [3] playing a key role [2]. Like other Codes of Nomenclature the scientific names of prokaryotes are treated as being Latin. While there is much freedom of choice in names at the rank of genus, subgenus, species and subspecies, scientific names above the rank of genus take a more restrictive, regulated form. Modern developments in the handling of the rapidly increasing data on taxa at different ranks have moved forward significantly in the past decades, with computer based systems storing and handling data. In that context the standardised use of the name *Acetobacter* would identify it as a genus name, but given the complexity of names at that rank accuracy would only be guaranteed against a database. The standardised form of the names *Acetobacter aceti* and *Acetobacter aceti* subsp. *liquefaciens* identifies these names as being at the rank of species and subspecies respectively. Names at the rank above genus and up to an including order are currently based on names at the rank of genus to which a standardised suffix is added (Table 1).

**Table 1:** Overview of the inter-relationship between the name of a genus, the suffix associated with a particular taxonomic rank and the resulting name based on Table 1 in the International Code of Nomenclature of prokaryotes [1]

Name at the rank of genus	Suffix	Rank	Resulting name
*Acetobacter*	*-inae*	Subtribe	*Acetobacterinae*
*Acetobacter*	*-eae*	Tribe	*Acetobactereae*
*Acetobacter*	*-oideae*	Subfamily	*Acetobacteroideae*
*Acetobacter*	*-aceae*	Family	*Acetobacteraceae*
*Acetobacter*	*-ineae*	Suborder	*Acetobacterineae*
*Acetobacter*	*-ales*	Order	*Acetobacterales*

The system has two advantages. The stem of the name of the genus can be systematically identified and it also serves as the nomenclatural type of the name at the higher rank. The suffixes –*inae*, -*oideae*, -*aceae*, -*ineae* and -*ales* (see Table 1 for the corresponding ranks) all allow unambiguous identification of the rank of the name. In the case of –*eae* this suffix is also part of the suffixes -*oideae*, -*aceae* and –*ineae*, indicating the longer the suffix the more accurate the assignment, since searching for the ending –*eae* would pick up the names of all ranks ending in –*eae*, including –*eae,* -*oideae*, -*aceae* and –*ineae*. In contrast searching for the endings -*oideae*, -*aceae* or –*ineae* would provide a unique suffix identifier for names at only one rank. The advantage of extending the principle of unique endings becomes apparent when applying this to names at the rank of subclass, class and phylum, especially when compared to the current system. When listing the full assignment to all taxonomic ranks for *Acetobacter* the NCBI (https://www.ncbi.nlm.nih.gov/Taxonomy/Browser/wwwtax.cgi?mode=Undef&id=434&lvl=3&keep=1&srchmode=1&unlock) lists:

Bacteria; Proteobacteria; Alphaproteobacteria; Rhodospirillales; Acetobacteraceae; Acetobacter.

and the GTBD (website https://gtdb.ecogenomic.org/) lists.

d__Bacteria; p__Proteobacteria; c__Alphaproteobacteria; o__Acetobacterales; f__Acetobacteraceae; g__Acetobacter;

The GTDB lists the ranks ie p__Proteobacteria (= phylum) or c__Alphaproteobacteria (= class) whereas the NCBI does not list the rank, but this is implied by the order of the names and to a limited extent by the suffixes. Clearly there are a number of problems. The use of the suffix –*ia* appears not only at the rank of domain, phylum and order, but is also a fairly common ending at the rank of genus. Although the current International Code of Nomenclature of Prokaryotes [1] does not cover names at the rank of phylum or domain (kingdom) it does define the names of classes as ending in –i*a*, and that they are in the plural and neuter gender. This is in contrast to most genus names ending in –*ia* that are considered to be feminine, singular words. Unfortunately for automatic text mining the gender is not normally indicated and makes automatic identification of the rank impossible unless one compares names against a reference database.

As indicated above the use of short suffixes is problematic if a shorter suffix is also part of a longer suffix (ie –*eae*) and in the case of –*ia*, it is used as endings for names at different ranks. Apart from the fact that the name *Proteobacteria* is a name used at the rank of class that is now used at the rank of phylum, the use of the names *Proteobacteria* and *Alphaproteobacteria* are descriptive names and provide no direct information on what the nomenclatural type may be. These names are the exception in the way there are formed, but names at the rank of class and subclass do not have to be formed from the name of a sub-ordinate taxon, while the nomenclatural type is one of the included orders that cannot automatically be inferred from the name itself ie.

*Proteobacteria*: not validly published name at the rank of phylum, nomenclatural type not defined.

*Alphaproteobacteria*: class nomenclatural type *Caulobacterales.*

*Rhodospirillales*: order, nomenclatural type *Rhodospirillum.*

*Acetobacteraceae:* family, nomenclatural type *Acetobacter*.

Tindall [4] has recently proposed a solution, whereby all names above the rank of genus should be formed from the name of a genus that by definition must be included in the higher taxon concerned and also serve as its nomenclatural type. Combined with a proposal to use –*aeota* or –*ota* [5, 6] as the suffix for names at the rank of phylum the use of a standardise suffix at the rank of phylum, class (-*ia*) and subclass (-*idae*) would give the names:

*Caulobacteraeota/ Caulobacterota* (phylum, nomenclatural type *Caulobacter*).

*Caulobacteria* (class, nomenclatural type *Caulobacter*).

*Caulobacteridae* (subclass, nomenclatural type *Caulobacter*).

However, the use of the name *Caulobacteria* as a simple text string ending in *–ia* would not distinguish it from a text string at the rank of genus ending in –*ia* ie *Ottowia* or *Owenweeksia*. If used at the rank of class the same ending is used, but is now plural and neuter rather than singular and feminine as is the case for the identically written names at the rank of genus (Table 2).

**Table 2:** The consequences of the names at the rank of genus, *Ottowia* and *Owenweeksia* being used as the stem of a name at the rank of class

Genus name	Gender etc	Stem	Class suffix	Resulting name at the rank of class	Gender etc
*Ottowia*	Singular, feminine	*Ottow-*	*-ia*	*Ottowia*	Plural, neuter
*Owenweeksia*	Singular, feminine	*Owenweeks-*	*-ia*	*Owenweeksia*	Plural, neuter

The situation with names at the rank of class is further complicated by the fact that names at the rank of class have come into use that were formed before a formal suffix was defined and includes names such as *Bacilli*, *Chlamydiae*, *Firmicutes* etc. Further misunderstanding appears to have arisen surrounding the proposals of Tindall [4] where Oren et al. [7] interpret the proposal as contravening Principle 3 “the scientific names of all taxa are Latin or latinized words treated as Latin regardless of their origin” [1]. However, the proposals of Tindall [4] clearly do not suggest that names should not be treated as Latin and the only point that seems to have been misunderstood is whether the text of Tindall [4] states that the suffix of the class name –*ia* is to be treated as Latin as a nominative plural in the feminine gender, rather than in the neuter gender. Tindall [4] “relegates” all suffixes to a table and makes no mention of either what those suffixes should be making it difficult to infer that the wording contravenes any part of the Code, let alone Principle 3. The utility of “relegating” the suffixes to a table is that it offers more flexibility when changes need to be made, rather than adding/deleting, changing text.

The issue of the suffixes of class names was discussed many years ago within the Judicial Commission [8, 9], where the suffix –*ia* was also considered, but also those ending in –*es* (*Actinomycetes* etc.). Alonso-Zarazaga [10] provides interesting insights into the use of both the gender of Latin nominative plural suffixes, but also breaks down rank specific suffixes into “connectors” and “endings”, coming to similar conclusions that some of the possible solutions duplicate endings used in the singular for names at the rank of genus. Using this principle for suffixes currently defined in the International Code of Nomenclature of Prokaryotes would give the following (Table 3):

**Table 3:** An overview of the principle outlined by Alonso-Zarazaga [10] where rank specific suffixes are divided into “connectors” and “endings” based on ranks and suffixes covered by the International Code of Nomenclature of Prokaryotes [1]. All endings are plural and with the exception of *–ia* are feminine

Suffix	Rank	Connector	Ending
*-inae*	Subtribe	*-in-*	*-ae*
*-eae*	Tribe	*-e-*	*-ae*
*-oideae*	Subfamily	*-oide-*	*-ae*
*-aceae*	Family	*-ace-*	*-ae*
*-ineae*	Suborder	*-ine-*	*-ae*
*-ales*	Order	*-al-*	*-es*
*-idae*	Subclass	*-id-*	*-ae*
*-ia*	Class	*none*	*-ia*

Clearly for class names the absence of a connector is one of the problems and plural, feminine gender endings –*ae*, -*es*, or –*us* are also not the solution. Given the “popularity” of –*ae* as a feminine plural suffix, the use of a connector such as –*inat*- would give the suffix –*inatae* that would provide a unique identifier for names at the rank of class. This would not require any alterations to the proposal of Tindall [4] that retain reference to the gender of names. While it would require making changes to all names that currently do not conform to either the formal suffix or being formed from the name of a genus ie *Bacilli* becomes *Bacillinatae* and *Alphaproteobacteria* would become *Caulobacteinatae,* the disruption would be of limited scope (ie affecting approximately 100 of the just under 20,000 currently validly published names). If the principle of endings that are plural in the feminine gender were to be followed then the suffix for phylum could be –*otae*. There is also some wisdom in the second alternative given by Tindall [4] and follows the principle used in the International Code of Nomenclature for algae, fungi and plants where no mention of gender is made, but suffixes defined.

Examples of texts discussing the use of standardised suffixes include Alonso-Zarazaga [10] and Naomi [11] and highlight the utility of such a system that is largely already in use in the International Code of Nomenclature of Prokaryotes. In a text mining and taxonomic context the use of a standardised suffix to denote the rank allows automatic assignment of rank of the name and forming all names from the genus name that is at the same time the nomenclatural type provides a useful, transparent system. However, the issues raised by Oren et al. [5] not only point to problems with properly interpreting texts, but also changes in the wording of The International Code of Nomenclature of Bacteria/Prokaryotes. Earlier versions, up to the 1966 [12] revision stated “Other considerations, such as absolute grammatical correctness, regularity, or euphony of names, more or less prevailing custom, regard for persons, etc., notwithstanding their undeniable importance, are relatively accessory,” while the post 1975 [13] revisions removed that text and now state “any name or epithet should be written in conformity with the spelling of the word from which it is derived and in strict accordance with the rules of Latin and Latinization”. The latter is a potential source of numerous problems where any name that is not “in strict accordance with the rules of Latin and Latinization” contravenes the Rules, is illegitimate and would need to be dealt with, hardly a recipe for nomenclatural stability as outlined in Principle 1. Perhaps it might be appropriate to re-instate the older wording that originated from the wording of the 1935 revision of the International Rules of Botanical Nomenclature and has been retained, with some degree of wisdom in the International Code of Nomenclature for algae, fungi and plants [14].
